# miRNAs and *NFKB1* and *TRAF6* target genes: The initial functional study in CD14+ monocytes in rheumatoid arthritis patients

**DOI:** 10.1590/1678-4685-GMB-2023-0235

**Published:** 2024-07-26

**Authors:** Isaura Isabelle Fonseca Gomes da Silva, Denise de Queiroga Nascimento, Alexandre Domingues Barbosa, Fabricio Oliveira Souto, Maria de Mascena Diniz Maia, Sergio Crovella, Paulo Roberto Eleuterio de Souza, Paula Sandrin-Garcia

**Affiliations:** 1 Universidade Federal de Pernambuco, Programa de Pós-Graduação em Genética e Biologia Molecular, Recife, PE, Brazil.; 2 Instituto Keizo Asami, Recife, PE, Brazil.; 3 Policlínica Jamacy de Medeiros, Cabo de Santo Agostinho, PE, Brazil.; 4 Universidade Federal de Pernambuco, Hospital das Clínicas, Recife, PE, Brazil.; 5 Universidade Federal de Pernambuco, Centro Acadêmico do Agreste, Caruaru, PE, Brazil.; 6 Universidade Federal Rural de Pernambuco, Departamento de Biologia, Recife, PE, Brazil.; 7 Universidade Federal de Pernambuco, Departamento de Genética, Recife, PE, Brazil.

**Keywords:** miRNA, NFKB1, TRAF6

## Abstract

We predicted miRNAs with regulatory impact on *NFKB1* and *TRAF6* gene expression and selected the miR-194-5p, miR-124-3p, miR-9-5p, and miR-340-5p and their target genes for expression analyses on CD14+ monocytes from rheumatoid arthritis (RA) patients and healthy controls. Additionally, we evaluated the influence of genes and miRNA expression on RA patients’ cytokine levels. No difference was observed in genes or miRNAs expression when compared to healthy controls and RA patients or clinical parameters. However, we found a significant difference between miR-194-5p and miR-9-5p levels (FC=-2.31; p=0.031; FC=-3.05;p=0.031, respectively) and non-prednisone users as compared to prednisone using patients. We conducted correlation analyses to identify the strength of the relationship between expression data and cytokine plasma levels. We observed a moderate positive correlation between miR-124-3p expression and IL-6 plasma levels (r=0.46; p=0.033). In addition, overexpression of miRNAs was concomitant to *TRAF6* and *NFKB1* genes as indicated by correlation analyses: *TRAF6* and miR-194-5p (r=0.60;p<0.001) and miR-9-5p (r=0.63;p<0.001) and *NFKB1* and miR-194-5p (r=0.72;p<0.001), miR-9-5p (r=0.72;p<0.001) and miR-340-5p (r=0.61;p<0.001). *NFKB1* and *TRAF6* genes and miRNAs monocyte expression do not appear to be related to RA but showed a significant difference in different groups of RA therapy. In addition, increased levels of miRNAs can be linked to concomitant overexpression of *TRAF6* and *NFKB1* in monocytes and act as its regulators.

## Introduction

Rheumatoid arthritis (RA) is a complex and autoimmune disease that affects joints and can promote irreversible disability in patients (Smolen et al., 2018). Genetic, epigenetic, and environmental factors can trigger RA. However, its etiology still needs to be fully understood. miRNAs can be related to RA trigger and its pathogenesis once it can influence gene regulation, thus leading to increased cytokines, chemokines, and autoantibodies and promoting tissue damage (de la Rica et al., 2013). 

In addition, until now, the role of innate immunity cells is not clear in the etiology or pathogenesis of the disease. Besides, evidence is emerging to support the influence of monocytes on RA (Evans et al., 2009; Stuhlmüller et al., 2010; Tsukamoto et al., 2017; Smiljanovic et al., 2018). Literature data indicates that biomarkers on monocytes and other immune cells could help to predict therapeutic responses in RA or indicate disease activity in other autoimmune diseases, such as Systemic lupus erythematosus (SLE) (Stuhlmüller *et al.*, 2010; Abd-Elhamid et al., 2017; Smiljanovic *et al.*, 2018). 

Transcriptome analysis of monocytes from RA patients indicated a dysregulation of inflammatory molecules when compared to osteoarthritis (OA) patients and healthy controls; among these, the NFκB pathway is the most important (Smiljanovic et al., 2018). The NFκB pathway is related to inflammation due to participation in the processes of activation, differentiation, and homeostasis of immune cells and secretion of pro-inflammatory cytokines (Sun et al., 2013; Liu et al., 2017). The NFκB family is a complex formed by two subunits (homodimer or heterodimer) composed of NFκB1, NFκB2, RelA, RelB, and c-Rel (Sun *et al.*, 2013; Liu *et al.*, 2017). The heterodimer composed of NFκB1/ RelA or NFκB1/c-Rel is predominant in different immune cells and plays a role in autoimmunity (Lawrence, 2009; Sun *et al.*, 2013). In addition, a key molecule for NFκB activation is the tumor necrosis factor receptor (TNFR)-associated factor 6 (TRAF6) (Walsh et al., 2015). TRAF6 is a cytoplasmatic adaptor protein responsible for promoting signal transduction induced mainly by TNFR and IL1R, which leads to the activation of the NFκB pathway (Walsh *et al.*, 2015). TRAF6 also mediates signals of various cellular receptors and acts in immunoregulatory functions, development, homeostasis, and activation of immune and non-immune cells (Wang et al., 2015a; Walsh *et al.*, 2015; Zhu et al., 2017).

The expression increase of TRAF6 and NFκB1 in RA patients synovium promotes a higher concentration of inflammatory cells in the joint and, consequently, tissue damage in the synovium (Zhu et al., 2012; Świerkot et al., 2016; Liu et al., 2017; Puchner et al., 2018). However, there is still no consensus on the monocyte’s participation in this TRAF6 and NFKB1 gene deregulation and which factors are responsible for this (Smolen et al., 2018). 

Some studies verified the interference of miRNAs on the regulation of the *NFKB1* and *TRAF6* genes in cancer (Huang et al., 2016; Wu et al., 2018; Zhu et al., 2020) and cardiovascular diseases (Liang et al., 2019). Specifically, in autoimmune diseases, only Yue et al. (2019) observed a negative regulation of *NFKB1* mediated by miR-9-5p in BV2 cells in the study with multiple sclerosis. Thus, cell assays using monocytes can allow new insights into the pathogenesis of RA and be particularly promising for personalized medicine in the disease (Evans et al., 2009; Davignon et al., 2013; Puchner et al., 2018). 

This case-control study aimed to verify potential miRNAs that target *TRAF6* and *NFKB1* genes and verify an association between *TRAF6* and *NFKB1* genes and miRNAs expression and RA etiopathogenesis and therapy. We also evaluated the expression of genes and miRNAs related to cytokine levels and the possible clinical significance of these findings in RA pathogenesis.

## Material and Methods

### Study participants

The present study is an observational case-control study. Twenty RA patients diagnosed according to the 2010 classification criteria of the American College of Rheumatology/European League Against Rheumatism (ACR/EULAR) (Smolen et al., 2010) at the Policlinica Doutor Jamacy de Medeiros and Hospital das Clinicas of Federal University of Pernambuco, Recife, Pernambuco, Brazil were enrolled in our study. Demographic data and clinical features were collected in appropriate questionnaires during clinical care. Biochemical analyses (C reactive protein (CRP), erythrocyte sedimentation rate (ESR), and rheumatoid factor (RF)) were evaluated from the peripheral blood of each individual participating in the study. Patients included were women naive for treatment or treated only with glucocorticoid and/or disease-modifying antirheumatic drugs (DMARDs) synthetic (methotrexate, leflunomide, sulfasalazine, or hydroxychloroquine). The RA patients who received any biological agents were excluded from the study. The RA patients were divided into three subgroups: naïve (untreated patients); monotherapy (RA patients on monotherapy of synthetic DMARDs (methotrexate, leflunomide or hydroxychloroquine)) and combined treatment (RA patients on combined therapy with methotrexate plus hydroxychloroquine or methotrexate plus sulfasalazine)) to evaluate the relation of the *TRAF6*, *NFKB1*, and miRNAs with the treatment strategies. Besides this, we also stratified RA patients according to the use of glucocorticoids (prednisone users and non-prednisone users).

The healthy control group consisted of 18 individuals matched by gender, age, BMI (Body Mass Index), and geographic region. We also performed biochemical analysis (ESR) and excluded individuals with a significant level of inflammation (ESR >30 mm/h) and with autoimmune, chronic, or infectious diseases. This study was approved by the Ethics Committee of the Health Sciences Center of the Federal University of Pernambuco (CAAE 10035418.4.0000.5208). All participants signed a written informed consent according to the Declaration of Helsinki.

### miRNA prediction analysis

The miRNAs were chosen using four prediction tools available online (TargetScan 7.1 (Agarwal et al., 2015); DIANA-MicroT (Paraskevopoulou et al., 2013); miRanda-mirSVR (Betel et al., 2010) and PicTar (Krek et al., 2005)). The miRNAs selected for the study were predicted at least in three tools and are more likely to have an impact on NFKB1 (Ensembl: ENSG00000109320) or TRAF6 (Ensembl: ENSG00000175104) gene expression.

### Cell Isolation

The peripheral blood mononuclear cell (PBMC) isolation was performed from peripheral blood collected in vacutainer tubes containing heparin according to standard density gradient centrifugation with Ficoll-Paque Plus (GE Healthcare, USA). Subsequently, 1 x 10^7^ cells were used from each individual to purify CD14+ monocytes using positive sorting with Dynabeads CD14 (Invitrogen, USA). Posteriorly, cells were labeled with anti-CD14-FITC (BD Biosciences, USA) and anti-CD3-PE-Cy5.5 (BD Biosciences, USA) and incubated for 30 min at 4 ºC to perform the immunofluorescence analysis in the flow cytometry Accuri C6 Flow Cytometer (BD Biosciences, USA).

### RNA isolation and determination of TRAF6 gene and miRNAs expression

The total RNA from monocytes was extracted using TRIzol^®^ reagent (Invitrogen, USA). The reverse transcription was performed by GoScript Reverse Transcription System (Promega, USA) according to the manufacturer’s protocol starting from 500 ng of RNA. We performed a quantitative reverse transcription PCR (qRT-PCR) using TaqMan probes as follows: NFKB1 (ID assay: Hs00765730), TRAF6 (ID assay: Hs00939742) GAPDH (ID assay: Hs03929097), ACTB (ID assay: Hs99999903), 18S (ID assay: Hs03003631). RPLP0 gene expression was also assessed using SYBR Green assay using 1X SYBR Green PCR Master Mix (Thermo Fisher Scientific, USA) and 10 µM of PCR primers previously validated (de Lima et al., 2019). *NFKB1* and *TRAF6* gene expression was normalized by GAPDH, ACTB, 18S, and RPLP0 reference genes.

The TaqMan MicroRNA Reverse Transcription Kit (Thermo Fisher Scientific, USA) was used to perform the cDNA of miRNAs analyzed using an input of 10ng of total RNA. Small RNAs analyses were performed with the probes miR-194-5p (ID assay: 000493), miR-124-3p (ID assay: 001182), miR-9-5p (ID assay: 000583), miR-340-5p (ID assay: 002258), RNU6B (ID assay: 001093) and RNU48 (ID Assay: 001006). miRNAs expression was normalized by RNU48 and RNU6B reference small RNAs. 

All analyses were performed in triplicate using the ABI Prism 7500 Sequence Detection System (Thermo Fisher Scientific, USA). The relative gene expression and miRNA expression were conducted following the Vandesompele method (Vandesompele et al., 2002) and MIQE guidelines (Bustin et al., 2009). 

*Cytokine levels*

Plasma samples were obtained from 25 individuals (16 RA patients and 9 healthy controls) during PBMC isolation after standard density gradient centrifugation and stored at -80 ºC until cytokine quantification. Cytokine levels (TNF-α, IL-6, IL-2, and IL-10) were measured using BD™ Cytometric Bead Array (CBA) Human Th1/Th2 Cytokine Kit II. All analyses were performed in an Accuri C6 Flow Cytometer (BD Biosciences, USA). 

### Statistical analyses

Data are expressed as mean ± standard deviation to quantitative variables or percentage and number to categorical variables. Relative expression levels were calculated using normalized data (Vandesompele et al., 2002). The normal distribution was tested according to the Shapiro-Wilk test, and parametric (ANOVA or T Student) or non-parametric (Kruskal-Wallis or Mann-Whitney *U*) tests were used as appropriate. Correlations between two continuous variables were measured using Pearson or Spearman’s correlation coefficient (*r*). P values < 0.05 were considered statistically significant. GraphPad Prism 6.0 software was employed for data analysis.

## Results

### Subjects of study

The demographic and clinical characteristics of RA patients and healthy controls are shown in Table 1. Both groups of patients and healthy controls were females with a mean age of 53.20±7.96 years and 53.83±4.54 years, respectively. RA group had a mean disease duration of 79.0 ±81.99 months and presented an active RA (DAS28-ESR: 5.41±1.20; CDAI: 28.99±12.97). Three patients were untreated, while nine patients received methotrexate, leflunomide, or hydroxychloroquine as monotherapy or in combination with prednisone, and eight patients were under treatment with combined synthetic DMARDs (methotrexate plus hydroxychloroquine or methotrexate plus sulfasalazine).

**Table 1 -:** Demographic data and clinical parameters features of patients with rheumatoid arthritis and healthy controls.

Variable	**RA patients (*N*=20)**	Healthy controls (N=18)
Age; mean ± SD years	53.20 ± 7.96	53.83 ± 4.54
BMI; mean ± SD	27.37 ± 5.91	26.28 ± 3.90
ESR, mean ± SD mm/h	29.40 ± 11.19	18.47 ± 7.42
CRP, mean ± SD mg/L	1.60 ± 2.09	
Age at RA onset; mean ± SD years	46.60 ± 9.48	
Disease duration, mean ± SD months	79.08 ± 81.99	
DAS28, mean ± SD	5.41 ± 1.20	
CDAI; mean ± SD	28.39 ± 12.97	
HAQ; mean ± SD	0.86 ± 0.73	
TJC; mean ± SD	11.05 ± 9.26	
SJC; mean ± SD	4.65 ± 4.73	
Rheumatoid factor positive^a^, *n* (%)	7 (53.85)	
Joint space narrowing presence^b^, n (%)	8 (80.00)	
Treatment		
Without treatment n (%)	3 (15.00)	
Corticosteroids, n (%)	11 (55.00)	
Methotrexate, n (%)	12 (60.00)	
Hydroxychloroquine, n (%)	9 (45.00)	
Leflunomide, n (%)	2 (10.00)	
Sulfasalazine, n (%)	1 (5.00)	
Hypertension	11 (55.00)	

^a^ ^-^ Data available for 13 patients; ^b-^ Data available for 10 patients; SD: Standard deviation; BMI: Body Mass Index; ESR: Erythrocyte sedimentation rate; CRP: C-reactive protein; DAS28: RA disease activity score 28 joint; CDAI: Clinical Disease Activity Index; HAQ: Health

Assessment Questionnaire; TJC: Tender joint count; SJC: Swollen joint count.

*miR-194-5p and miR-124-3p, miR-9-5p and miR-340-5p directly target the 3′UTR of TRAF6 and NFKB1 genes, respectively, according to in silico approach*

We identified 87 miRNAs predicted by DIANA-MicroT, 13 miRNAs predicted by TargetScan, 21 miRNAs predicted by miRanda-mirSVR, and 2 miRNAs predicted by PicTar for a potential binding site of TRAF6. We also considered different scores (DIANA-MicroT: miTG score; TargetScan: context score and Pct score; and miRanda-SVR: miSVR score) to indicate a possible impact on TRAF6 expression and selected the miR-194-5p and miR-124-3p to conduct the assays. Likewise, we found miRNAs with potential binding sites in *NFKB1* predicted by TargetScan (n=16), DIANA-MicroT (n=41), miRanda-mirSVR (n=17) and PicTar (n=1). The miR-9-5p and miR-340-5p were selected for analysis.

*TRAF6, NFKB1, and miRNAs expression are not related to development and clinical parameters but are influenced by the RA treatment strategy*

**Figure 1 -:**
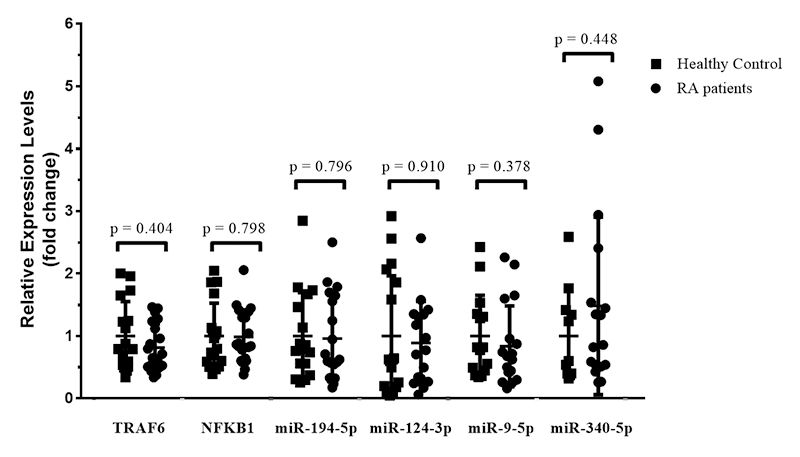
Relative expression levels of *TRAF6, NFKB1*, and miRNAs in monocytes of RA patients and healthy controls. The non-parametric Mann-Whitney test was used to test for statistical differences. Data are presented as mean (central line) and standard deviation (top and bottom of the line).

We evaluated the *TRAF6* and *NFKB1* genes and miRNAs expression in monocytes from RA patients and healthy controls. Relative expression data did not show a significant difference when comparing RA patients with healthy controls to both genes *TRAF6* (FC (Fold Change) = -1.227; p=0.404) and *NFKB1* (FC=-1.013; p=0.798). Likewise, miRNA expression data also did not show a significant difference when comparing RA patients with healthy controls for miR-194-5p (FC=-1.042; p=0.796), miR-124-3p (FC=-1.126; p=0.910), miR-9-5p (FC=-1.200; p=0.378) and miR-340-5p (FC=1.482; p=0.448). Data are shown in Figure 1.

In addition, we evaluated the correlations between genes and miRNAs expression with clinical characteristics of RA patients. We did not observe correlations between *TRAF6*, *NFKB1*, and miRNAs expression and disease duration, activity disease parameters (DAS28 and CDAI), or biochemical features (ESR and CRP). Furthermore, no significant correlation was found in the inflamed joint counts TJC (tender joints count), SJC (swollen joint count), or disability index, measured by HAQ. Correlation data are shown in Table 2. 

**Table 2 -:** Correlation between TRAF6, NFKB1 and miRNAs expression in CD14+ monocytes and clinical characteristics of RA patients.

Variables	NFKB1 gene		TRAF6 gene		miR-194-5p		miR-124-3p		miR-9-5p		miR-340-5p	
	r	p-value	r	p-value	r	p-value	r	p-value	R	p-value	r	p-value
Disease duration	0.18	0.450 ^b^	-0.02	0.944 ^b^	0.12	0.601 ^b^	-0.10	0.684 ^b^	0.28	0.262 ^b^	0.04	0.888 ^b^
DAS28	-0.23	0.321 ^a^	-0.14	0.567 ^b^	-0.29	0.208 ^b^	-0.20	0.448 ^a^	-0.14	0.578 ^b^	-0.29	0.254 ^b^
CDAI	-0.26	0.276 ^a^	-0.24	0.299 ^b^	-0.34	0.139 ^b^	-0.20	0.453 ^a^	-0.18	0.485 ^b^	-0.37	0.141 ^b^
HAQ	-0.15	0.516 ^b^	-0.30	0.195 ^b^	-0.13	0.571 ^b^	-0.37	0.145 ^b^	-0.11	0.671 ^b^	-0.32	0.204 ^b^
ESR	-0.18	0.458 ^a^	0.10	0.859 ^b^	-0.01	0.992 ^b^	0.28	0.270 ^a^	-0.28	0.253 ^b^	-0.11	0.664 ^b^
CRP	-0.19	0.517 ^b^	0.05	0.859 ^b^	-0.21	0.470 ^b^	0.31	0.359 ^b^	-0.29	0.357 ^b^	0.11	0.739 ^b^
TJC	-0.19	0.432 ^b^	-0.06	0.791 ^b^	-0.11	0.638 ^b^	-0.09	0.720 ^b^	0.04	0.874 ^b^	-0.18	0.485 ^b^
SJC	0.33	0.166 ^b^	0.15	0.530 ^b^	0.19	0.436 ^b^	-0.29	0.281 ^a^	0.11	0.671 ^b^	0.06	0.822 ^b^

r: correlation coefficient; DAS28: Disease Activity Score 28-joint; CDAI: Clinical Disease Activity Index; HAQ: Health Assessment Questionnaire; ESR: erythrocyte sedimentation rate; CRP: C-reactive protein; TJC: Tender joint count; SJC: Swollen joint count;

^a^ - Correlations was tested using Pearson’s correlation test; ^b^- Correlations was tested using Spearman’s correlation test.

Finally, we evaluated differences in the miRNA’s expression and RA therapy. RA patients were stratified according to the use of glucocorticoids (prednisone), and we observed that non-prednisone users showed significantly lower miR-194-5p expression when compared to prednisone users (FC=-2.31; p=0.031). Similarly, non-prednisone users showed lower miR-9-5p levels when compared to prednisone users (FC=-3.05; p=0.031) (Table 3). 

**Table 3 -:** Normalized quantitative expression of TRAF6 and NFKB1 genes and miRNAs expression in CD14+ monocytes from RA patients in different treatment strategies.

Variables	NFKB1 gene	TRAF6 gene	miR-194-5p	miR-124-3p	miR-9-5p	miR-340-5p
Treatment strategies						
Naïve (N = 3)	1.107 ± 0.198	1.423 ± 0.679	0.277 ± 0.120	0.66 (0.56-0.76)	0.14 (0.12 - 0.28)	0.130 ± 0.111
DMARDs as monotherapy (N = 9)	1.531 ± 0.484	1.632 ± 0.703	0.592 ± 0.351	0.47 (0.22 - 0.85))	0.40 (0.23 - 0.88)	0.270 ± 0.222
DMARDs combined (N = 8)	1.511 ± 0.869	1.578 ± 0.862	0.845 ± 0.704	0.50 (0.17 - 0.89)	0.31 (0.25 - 0.45)	0.782 ± 0.792
P-value	0.606 ^a^	0.961 ^a^	0.269 ^a^	0.756 ^b^	0.267 ^b^	0.102 ^a^
Use of glucocorticoid						
Prednisone user (N= 11)	1.656 ± 0.682	1.744 ± 0.793	0.84 (0.39 - 1.01)	0.36 (0.17 - 0.82)	0.48 (0.28 - 0.88)	0.631 ± 0.679
No Prednisone user (N = 9)	1.218 ± 0.509	1.377 ± 0.634	0.33 (0.19-0.36)	0.63 (0.38 - 0.87)	0.23 (0.14 - 0.40)	0.237 ± 0.338
P-value	0.128^a^	0.277 ^a^	**0.031** ^b^	0.228 ^b^	**0.031** ^b^	0.0941 ^a^

Normal distribution data was shown as mean ± standard deviation, while non-normal distribution data was shown as median (25th-75th). Naïve: RA patients untreated; DMARDs as monotherapy: RA patients treated with methotrexate, leflunomide, or hydroxychloroquine as monotherapy; DMARDs combined: RA patients treated with combined therapy (methotrexate plus hydroxychloroquine or methotrexate plus sulfasalazine). Bold means significant

^a^ Statistic was tested using parametric tests (Unpaired t-test or ANOVA); ^b^ Statistic was tested using non-parametric tests (Mann-Whitney or Kruskal-Wallis tests).

We also stratified RA patients according to their use of synthetic DMARDs: untreated (naive), monotherapy of synthetic DMARDs (methotrexate, leflunomide, or hydroxychloroquine), and RA patients with combined DMARDs (methotrexate plus hydroxychloroquine or methotrexate plus sulfasalazine). However, we did not observe significant differences among these groups.

*miR-124-3p expression in monocytes is positively correlated to IL-6 plasma levels*

We performed correlation analyses to verify the influence of *TRAF6* and *NFKB1* genes and miRNAs expression in the TNF-α, IL-6, IL-2, and IL-10 plasma cytokines levels. Correlation data are shown in Table 4. We observed a moderate positive correlation between miR-124-3p expression and IL-6 plasma levels (r=0.46 p=0.033). Besides this, the results indicate a tendency of correlation between *TRAF6* gene expression and IL-10 plasma levels (r = 0.40; p = 0.051). In addition, no significant correlation was observed between genes and miRNA expression and other plasma cytokine levels.

**Table 4 -:** Correlation between TRAF6, NFKB1 and miRNAs expression in CD14+ monocytes and TNF-α, IL-6, IL-2 and IL-10 plasma cytokines levels from RA patients and healthy controls.

Variables	NFKB1 gene		TRAF6 gene		miR-194-5p		miR-124-3p		miR-9-5p		miR-340-5p	
	r	p-value	r	p-value	r	p-value	r	p-value	R	p-value	r	p-value
TNF-α	0.12	0.556	0.14	0.515	-0.05	0.821	-0.05	0.824	0.04	0.859	-0.09	0.713
IL-6	0.19	0.373	0.25	0.247	0.20	0.367	**0.46**	**0.033**	0.01	0.984	-0.01	0.981
IL-2	0.31	0.134	0.23	0.276	0.33	0.125	-0.01	0.986	0.29	0.187	0.23	0.373
IL-10	0.34	0.093	0.40	0.051	0.17	0.441	0.08	0.719	-0.03	0.899	-0.2	0.951

r: correlation coefficient; BMI: Body Mass Index; DAS28: Disease Activity Score 28-joint; CDAI: Clinical Disease Activity Index; HAQ: Health Assessment Questionnaire; ESR: erythrocyte sedimentation rate; CRP: C-reactive protein; TJC: Tender joint count; SJC: Swollen joint count;

Correlations was tested using Spearman’s correlation test. Bold means significant.

*TRAF6 and NFKB1 gene expression are strongly correlated in monocytes*

**Figure 2 -:**
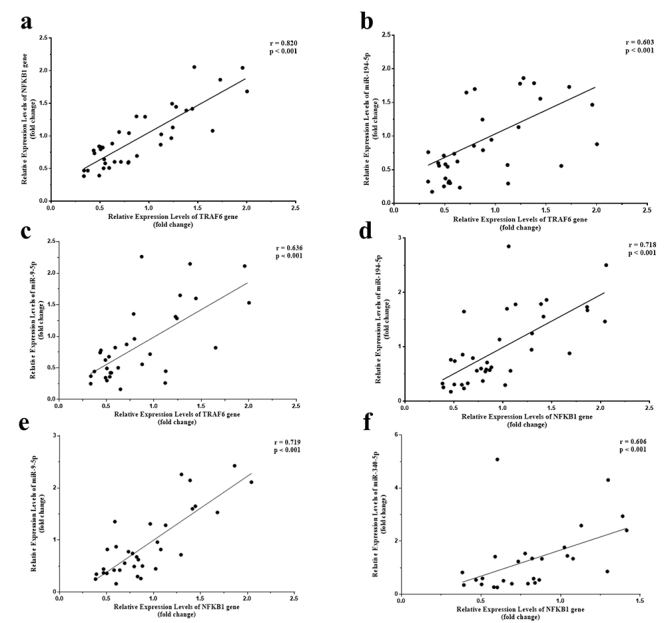
Scatter plot demonstrating correlations of mRNAs and miRNAs gene expression (fold change) in monocytes from RA patients and controls using Spearman’s correlation analyses. **a)** Correlation between *TRAF6* and *NFKB1* gene expression; **b)** Correlation between *TRAF6* gene expression and miR-194-5p expression; **c)** Correlation between *TRAF6* gene expression and miR-9-5p expression; **d)** Correlation between *NFKB1* gene expression and miR-194-5p expression; **e)** Correlation between *NFKB1* gene expression and miR-9-5p expression; **f)** Correlation between *NFKB1* gene expression and miR-340-5p expression.

*NFKB1* and *TRAF6* are closely related in many pathways, and we decided to assess whether the overexpression of *NFKB1* is concomitant to the overexpression of *TRAF6* in monocytes. Analysis using all individuals of the study (RA patients plus healthy controls) showed a strong positive correlation between *NFKB1* and *TRAF6* genes (r=0.820; p<0.001) (Figure 2a). In the same way, in the subgroup analyses, a strong positive correlation was observed in the RA patients’ group (r=0.836, p<0.001) and healthy control group (r=0.831, p<0.001), suggesting that both are related in the inflammatory and non-inflammatory context.

*miR-194-5p, miR-9-5p and miR-340-5p may act as regulators of TRAF6 and NFKB1 genes in monocytes*

We conducted correlation analyses to explore the potential role of miR-194-5p, miR-124-3p, miR-9-5p, and miR-340-5p in TRAF6 and NFKB1 gene expression. 

A significant correlation was observed between *TRAF6* gene expression and miR-194-5p, indicating a moderate positive correlation (r=0.603; p<0.001) (Figure 2b), which was maintained in the subgroups analysis (RA patients’ group: r=0.721; p<0.001; control group: r=0.521; p=0.041). A positive correlation was also observed between miR-9-5p and *TRAF6* gene (r=0.636; p<0.001) (Figure 2c) and in the subgroup analyses (RA patients’ group: r=0.552, p=0.018; control group: r=0.723, p=0.004). 

On the other hand, we did not find a correlation between *TRAF6* gene expression and miR-124-3p levels in the total individuals (r=-0.105; p=0.562) or the subgroups analyzed (RA patients’ group: r=0.154, p=0.553; control group: r=-0.159, p=0.556). Likewise, we did not observe a correlation between miR-340-5p and *TRAF6* gene expression in the analysis, including total individuals (r=0.455; p=0.149) or the subgroups analyzed (RA patients group: r=0.417, p=0.098 and control group: r=0.573, p=0.071).

Concerning *NFKB1*, the analysis using total subjects showed a strong positive correlation between *NFKB1* and miR-194-5p (r=0.718; p<0.001) (Figure 2d). Similarly, the subgroups analyzed showed the same findings (RA patients’ group: r=0.849; p<0.001; control group: r=0.642; p=0.006). We also observed a positive and strong correlation between *NFKB1* gene expression and miR-9-5p (r=0.719; p<0.001) (Figure 2e) and the analysis of the subgroups RA patients (r=0.719; p<0.001) or controls (r=0.750; p=0.002). Likewise, a positive correlation also was found between *the NFKB1* gene and miR-340-5p (r=0.606; p<0.001) (Figure 2f) and in the subgroup analyses (RA patients’ group: r=0.534, p=0.029; control group: r=0.727, p=0.014). On the other hand, the *NFKB1* gene expression and miR-124-3p were not correlated in the group of total individuals (r=-0.257; p=0.142) or the subgroups (RA patients’ group: r=-0.194, p=0.454; control group: r=-0.306, p=0.231).

Interestingly, our results also indicate a strong correlation between all the analyzed miRNAs, suggesting that their expression is similar in monocytes (miR-194-5p and miR-9-5p: r=0.819 p<0.001; miR-194-5p and miR-340-5p: r=0.661 p<0.001; miR-9-5p and miR-340: r=0.765 p<0.001).

## Discussion

In this study, we assessed four main topics related to *TRAF6* and *NFKB1* gene expression and its relationship with inflammation and RA. First, our study evaluated miRNAs with a possible effect on TRAF6 and NFKB1 expression through an *in silico* approach. Second, we conducted mRNA and miRNA expression analyses of RA patients and healthy controls in monocytes to assess an association with etiopathogenesis in RA. Third, we verified the influence of genes and miRNA expression on plasma cytokine levels. Fourth, we verified the correlation between mRNAs and miRNA expression.

Our results *in silico* showed that miR-9-5p and miR-340-5p seemed to be promising as regulatory factors of *NFKB1*, while miR-194-5p and miR-124-3p can potentially regulate *TRAF6* gene expression. In the Table S1 we summarize the main results of published studies on the expression of TRAF6 and NFKB1 and miR-194-5p, miR-124-3p, miR-9-5p, and miR-340-5p and its implication for Rheumatoid arthritis. 

Concerning *TRAF6* and *NFKB1* and miRNA expression in monocytes and RA development or clinical features, we did not observe differences in genes and miRNA expression between RA patients and healthy controls or about clinical features of RA patients (clinical activity indices, serological parameters, or treatment). Similar to our findings, no statistical differences were observed by Zhu et al. (2012) when evaluating the relationship between *TRAF6* expression in synovial tissue and clinical features of RA patients, although studies indicated overexpression of TRAF6 in synovial of RA and RA-fibroblast-like synoviocytes (RA-FLSs) (Zhu *et al.*, 2012; Wang et al., 2015b). The expression of the *NFKB1* gene seems to be relevant to RA severity, as observed by Sarmiento Salinas et al. (2018) since the authors reported an upregulation of *NFKB1* in active RA compared to inactive RA patients. Regarding miRNA expression, we also did not observe statistical differences in the development or clinical parameters of the RA patients. Low levels of miR-9-5p, miR-124-3p, and miR-340-5p in plasma or serum of RA patients as compared with controls were observed by Wang *et al.* (2015b); Goldbergová et al. (2018); Zhang et al. (2020). Likewise, it was observed by De la Rosa et al. (2020) in neutrophils from the peripheral blood of RA patients as compared to healthy controls. However, no relation was observed in RA severity or biochemical markers in either of these studies. On the other hand, Fernández-Ruiz et al. (2018) observed that miR-194-5p was overexpressed in whole blood of RA flare-up patients as compared to sustained remission RA. 

Interestingly, our observational study showed differences in miR-194-5p and miR-9-5p expression between non-prednisone users as compared to prednisone users, since patients using prednisone showed a 2.31-fold increase in levels of this miRNA, while miR-9-5p showed a 3.05-fold increase in its levels in prednisone users. The relation of miR-194-5p and treatment with prednisone was not tested previously. However, a study conducted by Fernández-Ruiz et al. (2018) failed to detect a relation between treatment with tofacitinib and miR-194-5p in RA patients. Moreover, to our knowledge, the miR-9-5p levels were not tested for any RA treatment strategies until now. Thus, we suggest that the expression of miR-194-5p and miR-9-5p in monocytes can be important to understand the effect of therapy in RA patients. Based on our findings, we suggest that prospective studies should be carried out to clarify whether there is a cause-and-effect relationship between therapy and miRNA expression.

Our findings also showed that miR-124-3p levels presented a significant positive correlation with IL-6 levels, while *TRAF6* expression showed a borderline correlation with IL-10 levels (p = 0.051). Similarly, miR-124a-3p overexpression promoted an upregulation of TNF-α, IL-6, and IL-1β production in the human cardiac myocyte (HCM) cell line (Liang et al., 2019). On the other hand, in the murine macrophage RAW264.7 cell line, the miR-124-3p levels were associated with decreased TNFα, IL-6, and IL-1β (Ma et al., 2014), while in the serum of RA patients, no significant correlation was found between miR-124-3p and IL-6, TNFα or IL-8 cytokine levels (Goldbergová et al., 2018). The miR-194-5p and miR-340-5p levels were related to a downregulation of TNF-α, IL-6, and IL-1β cytokine levels in mice nucleus pulposus cells and RA-fibroblast-like synoviocytes induced with lipopolysaccharides (Kong et al., 2018; Zhang et al., 2020) while miR-9-5p plasma levels of RA patients showed no correlation with IL-6, IL-1β and TNF-α levels (Wang et al., 2015b). Considering the discrepancies of different studies in different cells, we suggested that the influence of these genes and miRNAs expression on cytokine production depends on cellular type and it should be studied in each specific cellular context.

In addition, we verified the correlation between mRNAs and miRNAs expression. Our results showed a very strong positive correlation between *TRAF6* and *NFKB1* gene expression in monocytes. Both genes and their proteins act together to activate the NFκB pathway (Walsh et al., 2015; Liu et al., 2017) and probably show a co-expression to promote inflammation. It is important to note that the close relationship between both molecules can be explored in personalized medicine as a new approach to diseases that present dysregulation of TRAF6 or NFκB1. 

Considering that *TRAF6* and *NFKB1* genes were positively correlated in monocytes, we decided to assess the correlation of all miRNAs (miR-194-5p, miR-124-3p, miR-9-5p, and miR-340-5p) and both genes. We observed a strong positive correlation between *TRAF6* with miR-194-5p and miR-9-5p and also *NFKB1* with miR-194-5p, miR-9-5p and miR-340-5p. In contrast with our findings, studies showed that miR-194-5p plays a role in *TRAF6* suppression in mouse nucleus pulposus cells (Kong et al., 2018) and in THP-1 cells (Tian et al., 2015). In relation to *NFKB1*, Bazzoni et al. (2009) found similar results since the NFKB1 active by TLR4 enhanced miR-9-5p levels in human monocytes. On the other hand, Gu et al. (2016) also found the suppression of NFKB1 mediated by miR-9-5p in human primary chondrocytes of osteoarthritis patients. Regarding miR-340-5p, Li et al. (2016) found an *NFKB1* downregulation mediated by miR-340 in ovarian cancer cells. Our study and literature data agree that these miRNAs can act as regulators of the *TRAF6* and *NFKB1* genes, although this regulation appears to occur in different ways in different cell types. miRNAs commonly promote downregulation of target genes, but the opposite effect on specific cellular contexts has been seen (Vasudevan et al., 2007; Vasudevan, 2012; Ni and Leng, 2016; O’Brien et al., 2018). These studies suggested that miRNA can affect the mRNA translate activation, promoting mRNA activation to translation but not causing its degradation, which can lead to an increase in the number of mRNA molecules in a specific cellular type (Vasudevan, 2012; Ni and Leng, 2016). According to this, we suggested that these miRNAs (miR-194-5p, miR-9-5p, and miR-340-5p) bind in the 3’UTR region of their target genes (*TRAF6* and *NFKB1)* and block its translation through a mechanism in which the cell stores mRNAs ready for translation if needed. 

Although, in this observational study, a functional analysis between miRNAs and mRNAs was not performed, we provide insights that may encourage future studies to test this hypothesis. Besides this, some potential limitations exist, such as a reduced sample size to perform the analyses and a different number of participants in the cytokine levels measurement, which may affect our ability to recognize the real associations between data. Therefore, we encourage future studies, including studies with larger samples and prospective and functional studies that can test the hypotheses raised in our study.

In conclusion, our study showed that expression of the *TRAF6* and *NFKB1* genes and miRNAs in monocytes do not play a role in the development and pathogenesis of RA, although miR-194-5p and miR-9-5p levels showed differences in patients with different RA treatment strategy. In addition, we observed a significant correlation between genes and miRNAs analyzed and hypothesized the role of these miRNAs as regulators of *TRAF6* and *NFKB1* in monocytes. We suggest further studies to confirm whether there is a causality relationship between these findings.

## Supplementary material

The following online material is available for this article:

Table S1 - Results of the main published studies on expression of TRAF6 and NFKB1 genes and miR-194-5p, miR-124-3p, miR-9-5p and miR-340-5p its implication for Rheumatoid arthritis
