# Supplementary material for: miRNAs and NFKB1 and TRAF6 target genes: The initial functional study in CD14+ monocytes in rheumatoid arthritis patients
Source: Genet Mol Biol. 2024 Jul 26;47(2):e20230235. doi: 10.1590/1678-4685-GMB-2023-0235 (PMC11274900; doi:10.1590/1678-4685-GMB-2023-0235)
Supplement: Table S1 - [file 1415-4757-GMB-47-2-e20230235-s1.pdf]

Genetics and Molecular Biology
DOI: https://doi.org/10.1590/1678-4685-GMB-2023-0235

Supplementary Material to "miRNAs and *NFKB1* and *TRAF6* target genes: The initial functional study in CD14+ monocytes in Rheumatoid arthritis patients"

Table S1 - Results of the main published studies on expression of *TRAF6* and *NFKB1* genes and miR-194-5p, miR-124-3p, miR-9-5p and miR-340-5p its implication for rheumatoid arthritis.

Genes and miRNAs	Author(s) and Year	Type of sample	Findings
**TRAF6**	(Zhu *et al.*, 2012)	RA synovium	TRAF6 was overexpressed in RA synovium as compared to osteoarthritis and orthopedic arthropathies patients. It was observed a positive correlation between TRAF6 expression and synovitis score and inflammatory cell infiltration, although no significant difference in RA clinical features (DAS28-CRP, ESR, CRP, RF, ACPA, TJC, SJC, HAQ and erosion) was seen.
	(Wang H *et al.*, 2015)	Mouse collagen-induced arthritis (CIA)<br>Human RA- fibroblast-like synoviocytes (RA-FLSs)	TRAF6 was overexpressed in CIA joints as compared to normal DBA/1 mice and human RA-FLSs as compared to osteoarthritis-FLSs. In addition, TRAF6 inhibition by siTRAF6 reduced the severity of RA, joint inflammation and matrix metalloproteinase in mice. TRAF6 inhibition by Anti-TRAF6mAb in human RA-FLSs reduced IL-1β-stimulated migration, invasion and MMP-1, MMP-3 and MMP-9 secretion.
	(Zhang *et al.*, 2015)	RA- fibroblast-like synoviocytes (RA-FLSs)	Sinomenine treatment in RA-FLSs reduced gene and protein expression of TRAF6 and promotes reduction in RA-FLS proliferation, prevention of cartilage and subchondral bone destruction
	(Wu *et al.*, 2016)	WDFY3 transgenic Mice	WDFY3-deficient bone marrow-derived macrophages showed an increased levels of TRAF6 mRNA and protein as compared to bone marrow-derived macrophages wild type and the osteoclastogenesis in WDFY3-deficient cells is TRAF6 dependent.
	(Zhu *et al.*, 2017)	RA- fibroblast-like synoviocytes (RA-FLSs)	TRAF6 was overexpressed in RA-FLSs as compared to osteoarthritis-FLSs. In addition, TRAF6 inhibition by lentiviral transfection reduced secretion of cytokines (IL-1β, IL-8, IL-6, TNF-α) and matrix metalloproteinase (MMP-13, and MMP-3) and reduced RA-FLSs proliferation.
	(Brenke *et al.*, 2018)	Human peripheral blood mononuclear cells (PBMCs)<br>Mouse collagen-induced arthritis (CIA)	A small-molecule modulator (C25-140) reduced the TRAF6–Ubc13 protein interaction and promoted a reduction of TNF-α, IL-6 and IL-1β production in PBMCs and murine cells. In addition, in CIA model the treatment promoted ameliorated of arthritic index and symptoms of RA
	(Puchner *et al.*, 2018)	Mouse monocytes	TRAF6 (mRNA and protein levels) was overexpressed in RA non-classical monocytes subset (Ly6C-) compared with classical monocytes subsets (Ly6C+) in mice.

	Author(s) and Year	Type of sample	Findings
	(Liu *et al.*, 2019)	Mouse collagen-induced arthritis (CIA)	A histone modification (neddylation at Lys124) of TRAF6 gene regulates IL-17A-induced NF-κB activation.
	(Han *et al.*, 1998)	RA synovial tissue<br>Mouse collagen-induced arthritis (CIA)	NFκB1 was overexpressed in RA synovial tissue as compared to normal synovial tissue although no difference was seen when compared to osteoarthritis. In addition, the ability of NFκB1 to bind to DNA was increased in RA patients as compared to osteoarthritis and in mice CIA
	(Campbell *et al.*, 2000)	Mouse collagen-induced arthritis (CIA)	NFKB1 –/– mice showed a decrease of incidence and severity of arthritis in CIA. In addition, the synovial cells in acute arthritis showed a higher nuclear translocation of p50 (NFκB1 isoform)
	(Benito *et al.*, 2004)	RA synovial tissue	RA patients showed a overexpression of NFκB1 in cartilage-pannus junction as compared to synovial tissue of psoriatic arthritis and osteoarthritis
**NFKB1**	(Ahmed *et al.*, 2018)	Synovial membrane and articular cartilage of osteoarthritis (OA) patients	There was not significant differences in NFKB1 gene expression in articular cartilage and synovial membrane when compared OA patients and postmortem controls. In addition, there was not correlation between NFKB1 expression and *IL-8*, *IL-6*, or *MMP3* gene expression
	(Sarmiento Salinas *et al.*, 2017)	Leukocytes from peripheral blood	NFBK1 was upregulated in active RA compared as inactive RA and the use of nonsteroidal anti-inflammatory drugs also affect the expression.
	(Zhang *et al.*, 2018)	RA- fibroblast-like synoviocytes (RA-FLSs)	Aspirin reduces p-P50 (phosphorylated NFκB1 isoform) levels in RA-FLSs, however, remained unchanged levels of other NFκB1 isoforms (P50, p-P105 and P105). In addition, aspirin promoted apoptosis and inhibit the proliferation of RA-FLS.
	(Sabir *et al.*, 2019)	RA synovial tissue	NFBK1 was upregulated in RA synovial tissue as compared to healthy controls
	(Xu *et al.*, 2012)	human adipose derived stem cells (hASCs)<br>primary chondrocytes of osteoarthritis (OA)	MiR-194-5p downregulation enhanced the chondrogenic differentiation in human adipose derived stem cells (hASCs). In addition, miR-194-5p was overexpressed in IL-1β induced – primary chondrocytes of osteoarthritis patients.
	(Tian *et al.*, 2015)	THP-1 cell line	MiR-194-5p promotes a downregulation of *TRAF6* gene and TNF-α production in THP-1 cells stimulated by saturated fatty acid palmitic acid (PA)
**MiR-194-5p**	(Meng *et al.*, 2015)	whole blood of women with postmenopausal osteoporosis	MiR-194-5p was overexpressed in postmenopausal women with osteoporosis as compared to postmenopausal women with osteopenia or postmenopausal women with normal bone mineral density
	(Fernández-Ruiz *et al.*, 2018)	Whole blood of RA patients	miR-194-5p was overexpressed in RA flare-up patients as compared to sustained remission RA patients. In addition, treatment with tofacitinib does not affect miR194-5p expression.
	(Kong *et al.*, 2018)	mice nucleus pulposus cells	miR-194-5p was downregulated in nucleus pulposus cells induced by LPS and miR-194-5p mimics assay promotes a downregulation of inflammatory cytokines genes expression (TNF-α, IL-1β and IL-6), In addition, miR-194-5p overexpression reduced TRAF6 gene expression.

	Author(s) and Year	Type of sample	Findings
	(Shen *et al.*, 2019)	Whole blood of intervertebral disc degeneration (IDD) patients	miR-194-5p was downregulated in patients with intervertebral disc degeneration as compared to healthy controls
	(De la Rosa *et al.*, 2020)	Neutrophils of RA patients	miR-194-5p was downregulated in neutrophils from peripheral blood of RA patients as compared to healthy controls
**MiR-124-3p**	(Nakamachi *et al.*, 2009)	RA synoviocytes	MiR-124a-3p was downregulated in RA synoviocytes as compared to osteoarthritis synoviocytes and inhibited the proliferation of RA synoviocytes
	(Ma *et al.*, 2014)	peripheral leukocytes of patients with pulmonary tuberculosis<br>murine macrophage RAW264.7 cell line	MiR-124-3p is overexpressed in the peripheral leukocytes of patients with pulmonary tuberculosis as compared to controls and it downregulated TRAF6 expression in murine macrophage RAW264.7 cells. In addition, miR-124a-3p overexpression promoted a downregulation of IL-6 and TNF-α levels.
	(Qiu *et al.*, 2015)	Mice microglial cells (BV2 cells)<br>HEK293T cell line	MiR-124-3p overexpression promotes a decreased in TNF-α, IL-1β and IL-6 levels in BV2 cells. In addition, miR-124a-3p promotes a downregulation of TRAF6 gene and protein in HEK293T cells.
	(Nakamachi *et al.*, 2016)	adjuvant-induced arthritis (AIA) mice	miR-124a-3p promotes a inhibition of RA synoviocytes proliferation, leucocyte infiltration and cartilage or bone damage in adjuvant-induced arthritis (AIA) mice of RA synoviocytes, In addition, miR-124 decreased osteoclast differentiation in AIA mice.
	(Pávková Goldbergová *et al.*, 2018)	Serum of RA patients	MiR-124a-3p was downregulated in serum of RA patients as compared to controls and it was not correlated to clinical disease (DAS28, radiographic progression, autoantibodies ACPA and RF). In addition, miR-124-3p was not correlated to IL-6, TNF-a, IL-8, IL-13, IL-15 but it is correlated to MMP-3 levels.
	(Li *et al.*, 2018)	RA synovial fibroblasts	MiR-124a-3p was downregulated in RA synovial tissue as compared to osteoarthritis and joint trauma patients. In addition, miR-124a-3p inhibited the proliferation and invasion of RA synovial fibroblasts and promoted a downregulation in genes and proteins of MMP3, MMP13 and IL-1β.
	(Liang *et al.*, 2019)	human cardiac myocytes cell line	Overexpression of miR-124-3p promotes downregulation of *TRAF6* gene and upregulation of TNF-α, IL-6, and IL-1β production in human cardiac myocyte (HCM) cell line.
	(Wei *et al.*, 2019)	SW480 cell line	Overexpression of miR-124-3p promotes downregulation of *TRAF6* gene in SW480 cells.
**MiR-9-5p**	(Bazzoni *et al.*, 2009)	human monocytes and neutrophils	MiR-9-5p was overexpressed in human monocytes and neutrophils stimulated by LPS and induced the TNF-α and IL-1β production. In addition, the NFKB1 active by TLR4 enhanced miR-9-5p levels.
	(Guo *et al.*, 2009, p. 9)	Ovarian cancer cells	MiR-9-5p promoted a downregulation of mRNA and protein levels of NFKB1 in ovarian cancer cells.

	Author(s) and Year	Type of sample	Findings
	(Chen *et al.*, 2009)	Human primary peripheral blood monocytes	miR-9-5p was overexpressed in Human primary peripheral blood monocytes stimulated by oxidized low-density lipoprotein (oxLDL)
	(Wang W *et al.*, 2015)	Plasma of RA patients	MiR-9-5p was downregulated in plasma of RA patients as compared to healthy controls, systemic lupus erythematosus or Graves' disease. It was not observe correlation between miR-9-5p and RA severity (DAS28, SJC and TJC) or biochemical markers (CRP and ESR). In addition, it was observed a positive correlation between miR-9-5p plasma levels and TNF-α, IFN-γ, IL-17A, IL-4 and CXCL9 levels.
	(Magner *et al.*, 2016)	Whole blood of multiple sclerosis patients	MiR-9-5p was overexpressed in relapsing remitting multiple sclerosis (RRMS) patients as compared to healthy controls
	(Gu *et al.*, 2016)	human primary chondrocytes of osteoarthritis (OA)	MiR-9-5p is downregulated in knee osteoarthritis cartilage as compared to healthy controls. In addition, miR-9-5p downregulated NFKB1 and promotes chondrocytes proliferation
	(Oka, 2017)	Plasma of RA patients	MiR-9-5p levels is not associated to RA associated - interstitial lung disease (RA-ILD)
	(Wang *et al.*, 2017)	THP-1 derived macrophage cells<br>Human primary peripheral blood monocytes	MiR-9-5p overexpression promotes a downregulation of IL-1β and inflammasome activation in THP-1 derived macrophages stimulated by LPS, Alum or oxidized low-density lipoprotein (oxLDL). In addition, the human primary peripheral blood monocytes stimulated by oxLDL also promotes a downregulation of IL-1β and NLRP3 expression.
	(Yue *et al.*, 2019)	Mice microglial cells (BV2 cells)	A downregulation of miR-9-5p was observed in BV2 cells stimulated by LPS. In addition, the miR-9-5p overexpression in LPS-induced BV2 cells promotes a downregulation of NFKB1 and TNF-α and IL-6 production.
	(Li *et al.*, 2019)	Serum of RA patients	miR-9-5p was downregulated in RA-induced peripheral neuropathy as compared to RA patients without this condition
**MiR-340-5p**	(Ma *et al.*, 2016)	Mice Bone marrow macrophages<br>Mice OVX-induced osteoporosis	MiR-340-5p was downregulated during osteoclast differentiation
	(Li *et al.*, 2016)	Ovarian cancer cells	MiR-340-5p promoted a downregulation of mRNA and protein levels of NFKB1 in ovarian cancer cells.
	(De la Rosa *et al.*, 2020)	Neutrophils of RA patients	MiR-340-5p was downregulated in neutrophils from peripheral blood of RA patients as compared to healthy controls
	(Zhang *et al.*, 2020)	serum, synovial tissues, and fibroblast-like synoviocytes (FLSs) of RA patients	MiR-340-5p was downregulated in serum, synovial tissue and RA-FLSs as compared to healthy controls. In addition, microRNA-340-5p-mimic reduced the RA-FLSs proliferation, cytokines (TNF-a, IL-1b, IL-6 and IL-8) production in RA-FLSs

ESR: Erythrocyte sedimentation rate; CRP: C-reactive protein; DAS28: RA disease activity score 28 joint; CDAI: Clinical Disease Activity Index; HAQ: Health Assessment Questionnaire; TJC: Tender joint count; SJC: Swollen joint count.

References

Ahmed AS, Gedin P, Hugo A, Bakalkin G, Kanar A, Hart DA, Druid H, Svensson C and Kosek E (2018) Activation of NF-κB in synovium versus cartilage from patients with advanced knee osteoarthritis: A potential contributor to inflammatory aspects of disease progression. J Immunol 201:1918-1927.

Bazzoni F, Rossato M, Fabbri M, Gaudiosi D, Mirolo M, Mori L, Tamassia N, Mantovani A, Cassatella MA and Locati M (2009) Induction and regulatory function of miR-9 in human monocytes and neutrophils exposed to proinflammatory signals. Proc Natl Acad Sci U S A 106:5282-5287.

Benito MJ, Murphy E, Murphy EP, van den Berg WB, FitzGerald O and Bresnihan B (2004) Increased synovial tissue NF-kappa B1 expression at sites adjacent to the cartilage-pannus junction in rheumatoid arthritis. Arthritis Rheum 50:1781-1787.

Brenke JK, Popowicz GM, Schorpp K, Rothenaigner I, Roesner M, Meininger I, Kalinski C, Ringelstetter L, R'kyek O, Jürjens G *et al.* (2018) Targeting TRAF6 E3 ligase activity with a small-molecule inhibitor combats autoimmunity. J Biol Chem 293:13191-13203.

Campbell IK, Gerondakis S, O'Donnell K and Wicks IP (2000) Distinct roles for the NF-κB1 (p50) and c-Rel transcription factors in inflammatory arthritis. J Clin Invest 105:1799-1806.

Chen T, Huang Z, Wang L, Wang Y, Wu F, Meng S and Wang C (2009) MicroRNA-125a-5p partly regulates the inflammatory response, lipid uptake, and ORP9 expression in oxLDL-stimulated monocyte/macrophages. Cardiovasc Res 83:131-139.

De la Rosa IA, Perez-Sanchez C, Ruiz-Limon P, Patiño-Trives A, Torres-Granados C, Jimenez-Gomez Y, Del Carmen Abalos-Aguilera M, Cecchi I, Ortega R, Caracuel MA *et al.* (2020) Impaired microRNA processing in neutrophils from rheumatoid arthritis patients confers their pathogenic profile. Modulation by biological therapies. Haematologica 105:2250-2261.

Fernández-Ruiz JC, Ramos-Remus C, Sánchez-Corona J, Castillo-Ortiz JD, Castañeda-Sánchez JJ, Bastian Y, Romo-García MF, Ochoa-González F, Monsivais-Urenda AE, González-Amaro R *et al.* (2018) Analysis of miRNA expression in patients with rheumatoid arthritis during remission and relapse after a 5-year trial of tofacitinib treatment. Int Immunopharmacol 63:35-42.

Gu R, Liu N, Luo S, Huang W, Zha Z and Yang J (2016) MicroRNA-9 regulates the development of knee osteoarthritis through the NF-kappaB1 pathway in chondrocytes. Medicine (Baltimore) 95:e4315.

Guo LM, Pu Y, Han Z, Liu T, Li YX, Liu M, Li X and Tang H (2009) MicroRNA-9 inhibits ovarian cancer cell growth through regulation of NF-κB1: MiR-9 inhibits ovarian cancer cell growth. FEBS J 276:5537-5546.

Han Z, Boyle DL, Manning AM and Firestein GS (1998) AP-1 and NF-kappaB regulation in rheumatoid arthritis and murine collagen-induced arthritis. Autoimmunity 28:197-208.

Kong L, Sun M, Jiang Z, Li L and Lu B (2018) MicroRNA-194 inhibits lipopolysaccharide-induced inflammatory response in nucleus pulposus cells of the intervertebral disc by targeting TNF receptor-associated factor 6 (TRAF6). Med Sci Monit 24:3056-3067.

Li J, Song Q and Shao L (2018) MiR-124a inhibits proliferation and invasion of rheumatoid arthritis synovial fibroblasts. Eur Rev Med Pharmacol Sci 22:4581-4588.

Li P, Sun Y and Liu Q (2016) MicroRNA-340 Induces apoptosis and inhibits metastasis of ovarian cancer cells by inactivation of NF-kB1. Cell Physiol Biochem 38:1915-1927.

Li Z, Li Y, Li Q, Zhang Z, Jiang L and Li X (2019) Role of miR-9-5p in preventing peripheral neuropathy in patients with rheumatoid arthritis by targeting REST/miR-132 pathway. In Vitro Cell Dev Biol Anim 55:52-61.

Liang Y-P, Liu Q, Xu G-H, Zhang J, Chen Y, Hua F-Z, Deng C-Q and Hu Y-H (2019) The lncRNA ROR/miR-124-3p/TRAF6 axis regulated the ischaemia reperfusion injury-induced inflammatory response in human cardiac myocytes. J Bioenerg Biomembr 51:381-392.

Liu K, Chen K, Zhang Q, Zhang L, Yan Y, Guo C, Qi J, Yang K, Wang F, Huang P *et al.* (2019) TRAF6 neddylation drives inflammatory arthritis by increasing NF-κB activation. Lab Invest 99:528-538.

Ma C, Li Y, Li M, Deng G, Wu X, Zeng J, Hao X, Wang X, Liu J, Cho WC *et al.* (2014) microRNA-124 negatively regulates TLR signaling in alveolar macrophages in response to mycobacterial infection. Mol Immunol 62:150-158.

Ma Y, Shan Z, Ma J, Wang Q, Chu J, Xu P, Qin A and Fan S (2016) Validation of downregulated microRNAs during osteoclast formation and osteoporosis progression. Mol Med Rep 13:2273-2280.

Magner WJ, Weinstock-Guttman B, Rho M, Hojnacki D, Ghazi R, Ramanathan M and Tomasi TB (2016) Dicer and microRNA expression in multiple sclerosis and response to interferon therapy. J Neuroimmunol 292:68-78.

Meng J, Zhang D, Pan N, Sun N, Wang Q, Fan J, Zhou P, Zhu W and Jiang L (2015) Identification of miR-194-5p as a potential biomarker for postmenopausal osteoporosis. PeerJ 3:e971.

Nakamachi Y, Kawano S, Takenokuchi M, Nishimura K, Sakai Y, Chin T, Saura R, Kurosaka M and Kumagai S (2009) MicroRNA-124a is a key regulator of proliferation and monocyte chemoattractant protein 1 secretion in fibroblast-like synoviocytes from patients with rheumatoid arthritis. Arthritis Rheum 60:1294-1304.

Nakamachi Y, Ohnuma K, Uto K, Noguchi Y, Saegusa J and Kawano S (2016) MicroRNA-124 inhibits the progression of adjuvant-induced arthritis in rats. Ann Rheum Dis 75:601-608.

Oka S (2017) Plasma miRNA expression profiles in rheumatoid arthritis associated interstitial lung disease. BMC Musculoskelet Disord 18:7.

Pávková Goldbergová M, Lipková J, Fedorko J, Němec P, Gatterová J, Válková L, Ševčíková J and Vašků A (2018) Relationship of epigenetic variability of miR-124 to extracellular matrix remodelling and age-related MMP-3 expression in rheumatoid arthritis. Gen Physiol Biophys 37:703-710.

Puchner A, Saferding V, Bonelli M, Mikami Y, Hofmann M, Brunner JS, Caldera M, Goncalves-Alves E, Binder NB, Fischer A *et al.* (2018) Non-classical monocytes as mediators of tissue destruction in arthritis. Ann Rheum Dis 77:1490-1497.

Qiu S, Feng Y, LeSage G, Zhang Y, Stuart C, He L, Li Y, Caudle Y, Peng Y and Yin D (2015) Chronic morphine-induced microRNA-124 promotes microglial immunosuppression by modulating P65 and TRAF6. J Immunol 194:1021-1030.

Sabir JSM, El Omri A, Banaganapalli B, Al-Shaeri MA, Alkenani NA, Sabir MJ, Hajrah NH, Zrelli H, Ciesla L, Nasser KK *et al.* (2019) Dissecting the role of NF-κb protein family and its regulators in rheumatoid arthritis using weighted gene co-expression network. Front Genet 10:1163.

Sarmiento Salinas FL, Santillán Benítez JG, Hernández Navarro MD and Mendieta Zerón H (2017) NF-κB1/IKKε gene expression and clinical activity in patients with rheumatoid arthritis. Lab Med 49:11-17.

Shen L, Xiao Y, Wu Q, Liu L, Zhang C and Pan X (2019) TLR4/NF-κB axis signaling pathway-dependent up-regulation of miR-625-5p contributes to human intervertebral disc degeneration by targeting COL1A1. Am J Transl Res 11:1374-1388.

Tian H, Liu C, Zou X, Wu W, Zhang C and Yuan D (2015) MiRNA-194 regulates palmitic acid-induced toll-like receptor 4 inflammatory responses in THP-1 Cells. Nutrients 7:3483-3496.

Wang H, Chen W, Wang L, Li F, Zhang C and Xu L (2015) Tumor necrosis factor receptor-associated factor 6 promotes migration of rheumatoid arthritis fibroblast-like synoviocytes. Mol Med Rep 11:2761-2766.

Wang W, Zhang Y, Zhu B, Duan T, Xu Q, Wang R, Lu L and Jiao Z (2015) Plasma microRNA expression profiles in Chinese patients with rheumatoid arthritis. Oncotarget 6:42557-42568.

Wang Y, Han Z, Fan Y, Zhang J, Chen K, Gao L, Zeng H, Cao J and Wang C (2017) MicroRNA-9 inhibits NLRP3 inflammasome activation in human atherosclerosis inflammation cell models through the JAK1/STAT signaling pathway. Cell Physiol Biochem 41:1555-1571.

Wei C, Lei L, Hui H and Tao Z (2019) MicroRNA-124 regulates TRAF6 expression and functions as an independent prognostic factor in colorectal cancer. Oncol Lett 18:856-863.

Wu DJ, Gu R, Sarin R, Zavodovskaya R, Chen CP, Christiansen BA, Zarbalis KS and Adamopoulos IE (2016) Autophagy-linked FYVE containing protein WDFY3 interacts with TRAF6 and modulates RANKL-induced osteoclastogenesis. J Autoimmun 73:73-84.

Xu J, Kang Y, Liao WM and Yu L (2012) MiR-194 regulates chondrogenic differentiation of human adipose-derived stem cells by targeting Sox5. PLoS One 7:e31861.

Yue P, Jing L, Zhao X, Zhu H and Teng J (2019) Down-regulation of taurine-up-regulated gene 1 attenuates inflammation by sponging miR-9-5p via targeting NF-κB1/p50 in multiple sclerosis. Life Sci 233:116731.

Zhang HC, Liu MX, Wang EP, Lin Z, Lv GF and Chen X (2015) Effect of sinomenine on the expression of rheumatoid arthritis fibroblast-like synoviocytes MyD88 and TRAF6. Genet Mol Res 14:18928-18935.

Zhang S, Meng T, Tang C, Li S, Cai X, Wang D and Chen M (2020) MicroRNA-340-5p suppressed rheumatoid arthritis synovial fibroblast proliferation and induces apoptotic cell number by targeting signal transducers and activators of transcription 3. Autoimmunity 53:314-322.

Zhang X, Feng H, Du J, Sun J, Li D, Hasegawa T, Amizuka N and Li M (2018) Aspirin promotes apoptosis and inhibits proliferation by blocking G0/G1 into S phase in rheumatoid arthritis fibroblast-like synoviocytes via downregulation of JAK/STAT3 and NF-κB signaling pathway. Int J Mol Med 42:3135-3148.

Zhu LJ, Dai L, Zheng DH, Mo YQ, Ou-Yang X, Wei XN, Shen J and Zhang BY (2012) Upregulation of tumor necrosis factor receptor-associated factor 6 correlated with synovitis severity in rheumatoid arthritis. Arthritis Res Ther 14:R133.

Zhu LJ, Yang TC, Wu Q, Yuan LP, Chen ZW, Luo MH, Zeng HO, He DL and Mo CJ (2017) Tumor necrosis factor receptor-associated factor (TRAF) 6 inhibition mitigates the pro-inflammatory roles and proliferation of rheumatoid arthritis fibroblast-like synoviocytes. Cytokine 93:26-33.
